# The Advent of Molecular Targeted Therapies Against Cancer. Toward Multi‐Targeting Drugs Through Materials Engineering: A Possible Future Scenario

**DOI:** 10.1002/smsc.202400113

**Published:** 2024-05-28

**Authors:** Marianna Puzzo, Marzia De Santo, Catia Morelli, Antonella Leggio, Luigi Pasqua

**Affiliations:** ^1^ Services Department Laboratory of Clinical, Biomolecular and Genetic Analysis Hospital Facility Annunziata 87100 Cosenza Italy; ^2^ Department of Pharmacy, Health and Nutritional Sciences University of Calabria via P. Bucci 87036 Arcavacata di Rende (CS) Italy; ^3^ NanoSiliCal Devices srl Via P. Bucci 44A 87036 Arcavacata di Rende (CS) Italy; ^4^ Department of Environmental Engineering University of Calabria via P. Bucci 87036 Arcavacata di Rende (CS) Italy

**Keywords:** mesoporous silica nanoparticles, molecular multi‐targeting nanostructured device, molecular targeted therapy, nanomedicine, nanostructured therapeutics, personalized medicine

## Abstract

The authors, actively engaged in the development of mesoporous silica‐based solutions, initially for modified drug release, later for the smart administration of conventional chemotherapeutic cytotoxic drugs, present the evolution of the concept of targeted therapy across different disciplines. They also discuss the diverse therapeutic needs and related challenges (adverse drug effects) that have unfolded over the last 30 years. Nanomedicine potentialities, mainly against cancers, that have emerged globally during the intense research activity of the last few decades, are critically discussed. The authors glimpse the growing potential of immune‐based therapeutic solutions, including those assisted by nanotechnology, as well as molecular targeted therapies (MTT) on which they focus. The advantages offered by targeted molecular therapies, despite the limits of monotargeted therapies, suggest the engineering of multi‐targeted therapies. Nanomedicine solutions such as ligand‐specific internalization and pH‐sensitive drug release that they have extensively tested and recently presented in open literature, still remain available instruments. According to the authors, MTT can offer shining perspectives in the near future that will depend on a thorough comprehension of nanostructures synthesis and tumor physiology. This article gives an interdisciplinary point of view tailored for non‐specialist readers imagining possible future scenarios in the field.

## Graphical abstract

Schematic, non‐exhaustive representation of possible approaches in molecular targeted cancer therapies. Single‐, dual‐, and multi‐targeting drugs, both real and perspectively imagined (Molecular multi‐targeting nanodevices), are represented. Simultaneous targeting of multiple pathways enhances synergistic effects against cancer progression. Materials engineering allows the development of nanostructured devices to deliver drugs simultaneously to multiple targets, enabling precise and efficient therapeutic interventions.

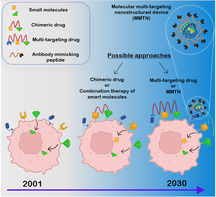

## 1 Introduction

Initially dedicated to the development of nanostructured particle‐based drug delivery systems, the authors later shifted their focus to the design of stimuli‐responsive drug‐targeting nanostructured devices.

In this article, they present their viewpoint on the evolution over time of the needs and technical requirements for achieving optimal outcomes, both in terms of efficacy and the reduction of toxicity, when administering anticancer drugs to patients.

The evolution of these needs can be considered to be overlapping with the advancement of the concept of cancer‐targeted therapy. Starting with methotrexate and 5‐fluorouracil, advancing through tyrosin kinase inhibitors (TKI) and hormone therapy,^[^ ^1^ ^]^ current targeted therapy is oriented toward the use of multi‐targeting drugs.

In general terms, an oncologic therapy can be considered targeted when a drug blocks tumor‐specific metabolic pathways. Specifically, as defined by Sledge,^[^ ^2^ ^]^ “…should attack a biologically important process central to a hallmark of cancer”. In accordance with this definition, as previously anticipated, all TKI as well as methotrexate, 5‐fluorouracil^[^ ^3^ ^]^ and drugs used in hormone therapy, can be considered as targeted therapies.

As reported by Peters,^[^ ^1^ ^]^ the National Cancer Institute (NCI) defined targeted therapy in 2012 as “a treatment that uses drug to attack specific cancer cells”. Subsequently, this definition evolved to encompass various mechanisms of action, including the inhibition of specific enzymes or proteins crucial for the growth of cancer cells, as well as the enhancement of the immune system's capability to eliminate cancer cells. Furthermore, the possibility of specifically attacking cancer cells through alternative routes, such as the use of small‐molecule drugs or monoclonal antibodies, has been introduced.

Cancer arises from somatic alterations that drive its growth and metastasis. It's important to note that each cancer type can be associated with different somatic changes. Consequently, individuals diagnosed with the same type of cancer undergoing the same therapeutic treatment may exhibit different responses if their cancers are characterized by diverse mutations.

Few words are able to give us a striking evidence of the urgent need to align anticancer therapies to the somatic mutations that drive cancer, even when distinct mutations yield the same cancer. The diagnostic assessment, pivotal for shaping anticancer strategies, advances at a higher level specifically focusing on the genetic speciations of the mutations driving cancer.

In essence, each cancer is characterized by a distinct panel of somatic changes that define the mechanisms driving its development. Acquiring knowledge about these changes is essential for identifying the right therapeutic treatment. Consequently, the precision medicine approach, in all the cases in which it is applied, represents a personalized medicine strategy. The targets involved, in fact, are related to the somatic mutations that significantly contribute to defining the prognosis.

This significant methodological advancement and the planning of anticancer therapeutic approaches based on the genetic speciation of tumor, have already started.

According to our opinion, within the current landscape, most promising developments in oncological therapies will predominantly emerge in the fields of immuno‐based, also nano‐assisted treatments. Relevant advancements, on the other side, will concern the therapeutic solutions characterized by molecular targeted therapies (MTT) and possible joint solutions among them and immunology. Immunology is beyond the scope of this analysis article so our discussion will concern therapeutic solutions involving MTT, both broadly and in perspective. We will particularly explore the potentialities offered by nanostructured skeleton‐based multivalent solutions, candidates to enhance prognosis and address persistent drug resistance issues.

## 2 The Beginnings of Targeted Therapies

Conventional chemotherapies primarily act on rapidly dividing tumor cells by impeding the survival of actively proliferating cells by blocking DNA and RNA synthesis, hindering mitosis, and/or forming covalent bonds with DNA, RNA, and proteins.^[^ ^4^ ^]^ 5‐fluorouracil (5‐FU) is a cytotoxic agent, and its mechanism of action involves inhibiting thymidylate synthase (TS). Due to this targeted action on a specific enzyme involved in DNA synthesis, 5‐FU can be considered a targeted chemotherapeutic agent.

First synthesized by Heidelberger and colleagues in 1957,^[^ ^3^ ^]^ 5‐FU is one of the earliest compounds with proven antitumor activity. It remains a widely used chemotherapy agent, particularly as a first‐line treatment for various epithelial malignancies, including stomach, colon, head and neck, and breast carcinomas.

5‐FU is a fluoropyrimidine (FpD), an uracil analogue wherein a fluorine atom replaces the hydrogen at the 5′ carbon position. As an antimetabolite, 5‐FU is a molecule that shares a similar structure with other naturally occurring molecules in the body but performs distinct functions. This peculiarity enables it to interfere with the growth and division of tumor cells. 5‐FU was developed from the initial observation that tumor tissues exhibit a faster uptake of uracil compared to normal tissues.^[^ ^5^ ^]^ Once inside the cells, the FpD is misincorporated into RNA and DNA in place of uracil or thymine and disrupting their normal functions.

**Figure 1:**
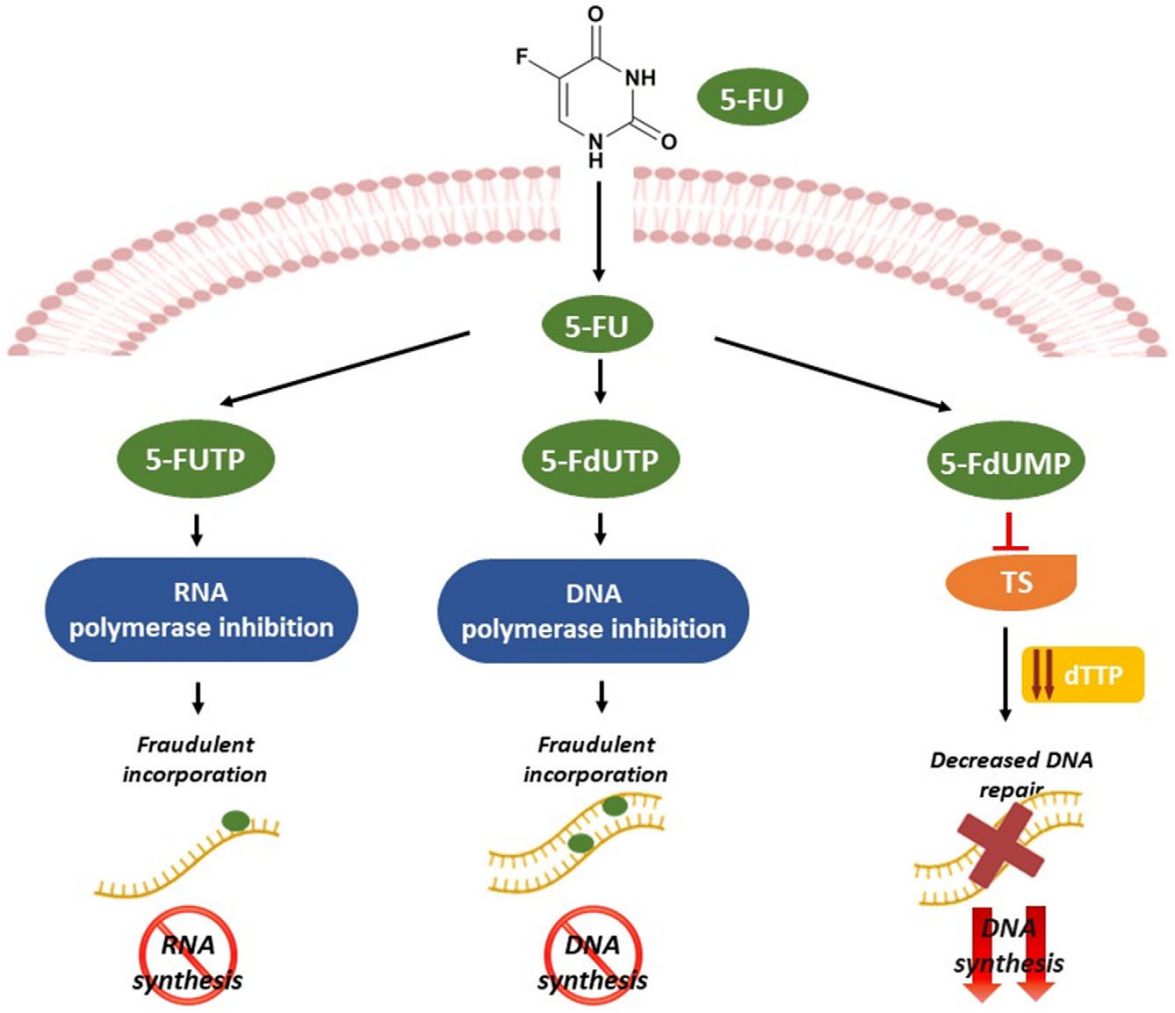
Schematic representation of 5‐FU mechanism of action. 5‐FU metabolites interfere with cell growth by acting at three distinct levels: 1) fluorouridine triphosphate (FUTP) is misincorporated into RNA, causing alterations in RNA processing and function; 2) 5‐fluorodeoxyuridine triphosphate (FdUTP) can affect DNA synthesis by directly incorporating into DNA; 3) 5‐Fluorodeoxyuridine monophosphate (FdUMP) inhibits thymidylate synthase (TS), interfering DNA repair and replication.

In addition, the active metabolite of 5‐FU, fluorodeoxyuridine monophosphate (FdUMP), acts as an irreversible inhibitor of thymidylate synthase (TS) leading to deoxythymidine triphosphate (dTTP) depletion, impairing the DNA replication and repair processes. Consequently, this disruption can trigger apoptosis, providing a targeted strategy to impede tumor progression.^[^ ^6^ ^]^ (See **Figure** **1**).

As it generally occurs with chemotherapy agents, also 5‐FU, is not devoid of off‐target toxicities, which might depend on the administration route employed. Indeed, myelotoxicity is the major side effect in patients receiving bolus doses, while stomatitis, palmar‐plantar erythrodysesthesia (hand‐foot syndrome), and neuro‐ and cardio‐toxicity are often associated with continuous infusions. Other effects, such as diarrhea, nausea and vomiting, dermatitis and alopecia occur during both continuous infusion and bolus‐dose regimens.^[^ ^7^ ^]^ Folate metabolism, NF‐kβ signaling, dihydropyrimidine dehydrogenase (DYPD) deficiency, and cell responses to stress seem to be the main pathways involved in 5‐FU mediated toxicity.^[^ ^8^ ^]^

Indeed, in the clinical management of these setting patients, the therapeutic plan as well as the therapeutic 5‐FU dose are established based on the presence of polymorphic variants present on DPYD gene sequence.^[^ ^9^ ^]^ Finally, patients’ response to 5‐FU therapy is very often compromised by the development of resistance, whose causes are multi‐factorial and include DNA mismatch repair deficiency, altered metabolism which impairs FdUMP production, high TS expression and/or activity or dysregulated programmed cell death.

Methotrexate (MTX), also known as amethopterin, is a cytotoxic agent employed in the treatment of diverse cancer types. It functions as a folate antagonist by inhibiting dihydrofolate reductase and is recognized as a cornerstone in anti‐folate therapy for several malignancies.^[^ ^10^ ^]^

**Figure 2:**
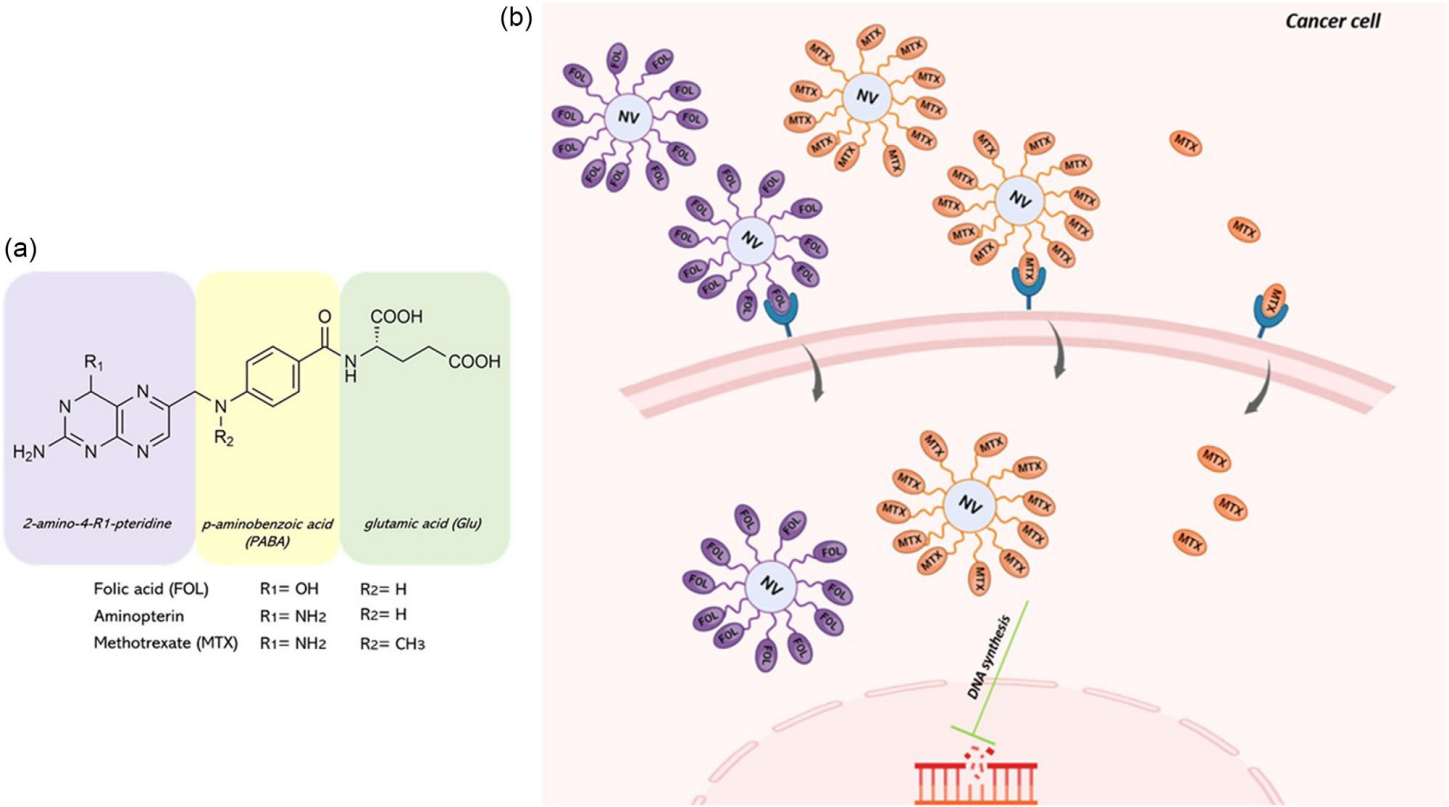
a) Chemical structure of folate family; b) MTX activity as both receptor‐specific ligand targeting folic acid receptor (FAR), similarly to how folic acid operates, and a cytotoxic agent inhibiting dihydrofolate reductase, thereby disrupting DNA synthesis and impeding cell proliferation.

MTX is a synthetic organic compound and, as anti‐folate drug, its entry into the cells can be mediated by two main transport systems: the reduced folate carriers (RFC) and membrane associated folate acid receptors (FAR).^[^ ^11^ ^]^ Originating in the late 1940s, MTX was developed as a less toxic derivative of aminopterin (**Figure** **2**a), a folic acid antagonist employed in the treatment of acute leukemia in children. Its therapeutic effect is attributed primarily to the inhibition of human dihydrofolate reductase (DHFR), an enzyme localized in the cytoplasm. Various delivery platforms for targeted cytotoxic therapy have been developed through the conjugation of a targeting ligand on their external surfaces, along with additional functionalizations to transport therapeutic and/or imaging payloads for intracellular delivery. Folic acid (FOL) has been extensively employed as a targeting ligand, given that the receptor for folic acid presents a useful target for tumor‐specific drug delivery for several reasons.^[^ ^12^ ^]^

MTX exhibits a potential activity as receptor‐specific ligand toward FAR due to its high structural similarity to FOL (Figure 2b).

**Figure 3:**
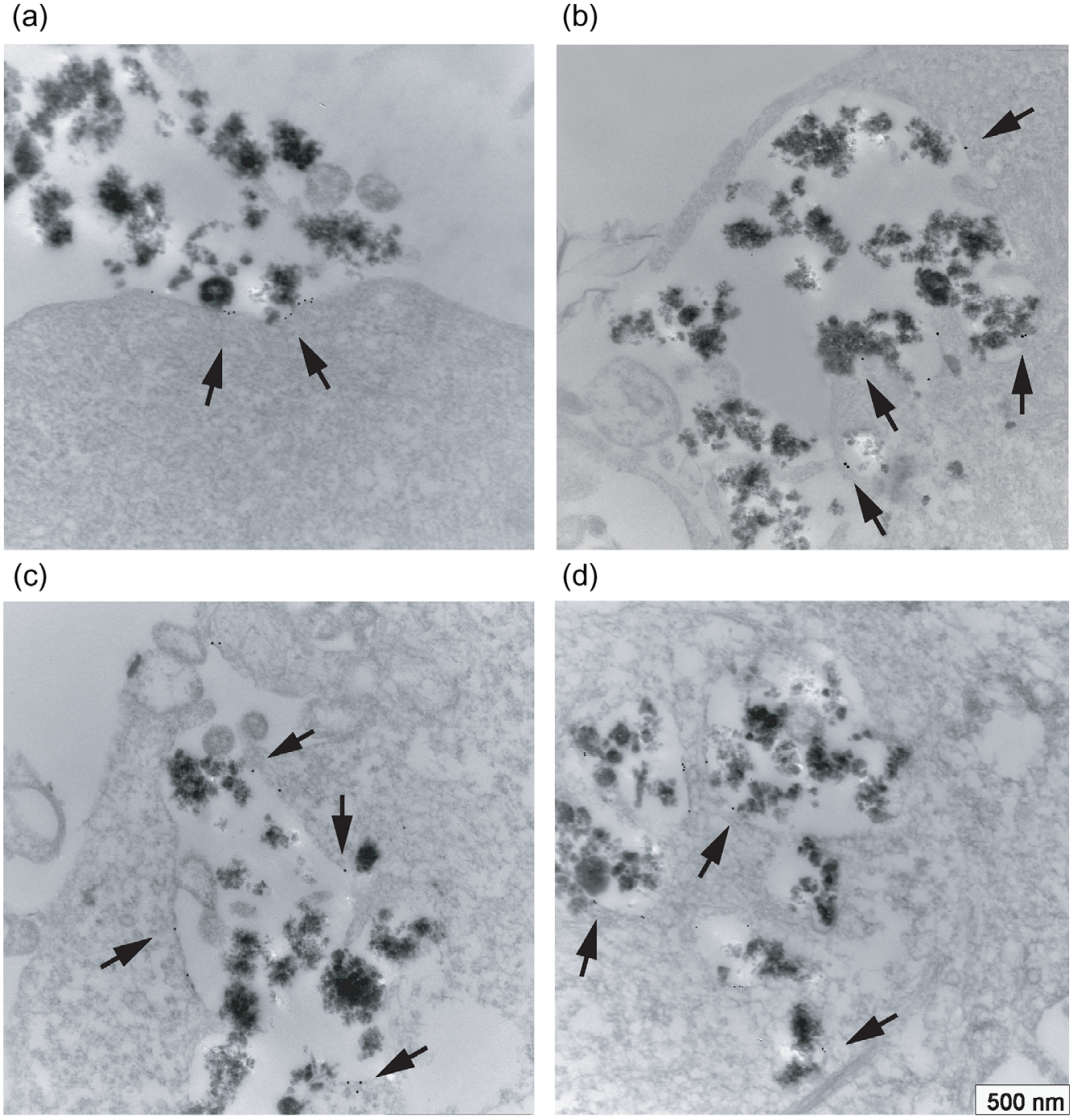
Immunogold labelling for FR in HeLa cells treated with FOL‐MSNs for 1 h. a) FOL‐MSNs approach a membrane region enriched in FR clusters, b) are engulfed through folic acid recognition by FR, c) are internalized by the cell and d) co‐localize with FR in the endocytosis vesicles. Reproduced with permission.^[^ ^18^ ^]^ 2011, The Royal Society of Chemistry.

MTX has been employed as a dual‐acting single molecule that functions as both a targeting ligand to a cancer‐specific receptor and as a therapeutic agent that induces cytotoxic effects upon internalization into the cells.^[^ ^13^ ^]^ In 2007 our group pioneered the development of folic acid functionalized mesoporous silica nanoparticles (FOL‐MSNs) loaded with cisplatin inside the pores (FOL‐MSN‐CP).^[^ ^14^ ^]^ A detailed study of the active internalization process in FR overexpressing cancer cells successively has shown that MSNs do not enter cells unless opportunely functionalized and that cellular uptake depends on the specific function(s) grafted on the MSNs external surface. Indeed, a highly specific, FR‐mediated, cellular internalization of FOL grafted MSNs (FOL‐MSNs), occurs exclusively in FR expressing cancer cells (**Figure** **3**), while no uptake was observed in FR negative normal cells. Interestingly, in agreement with other authors’ observations,^[^ ^15^, ^16^, ^17^ ^]^ also fluorescein grafted MSNs (MSN‐FITC) are able to enter cells through a nonspecific, caveolae‐mediated, endocytosis.^[^ ^18^ ^]^ Therefore, we wouldn't relay on MSN‐FITC diagnostic use (e.g., in cancer imaging), although they could still passively accumulate at the tumor site by means of the enhanced permeability and retention (EPR) effect.^[^ ^19^ ^]^ In this context, it must be underlined that the internalization process strongly depends on the nature, size and morphology, and surface charge of the tested particles as well as on the cell types and culture conditions. For instance, particles of 1 μm and over are able to trigger phagocytosis by the reticuloendothelial system (RES), while the uptake of smaller particles (<300 nm) generally occurs through different endocytosis pathways (e.g., receptor‐mediated, caveolin‐dependent, clathrin‐dependent as well as clathrin‐ and caveolin‐independent endocytosis).^[^ ^18^, ^20^, ^21^, ^22^, ^23^ ^]^

## 3 Engineered Nanodevices for Targeted Chemotherapy

**Figure 4:**
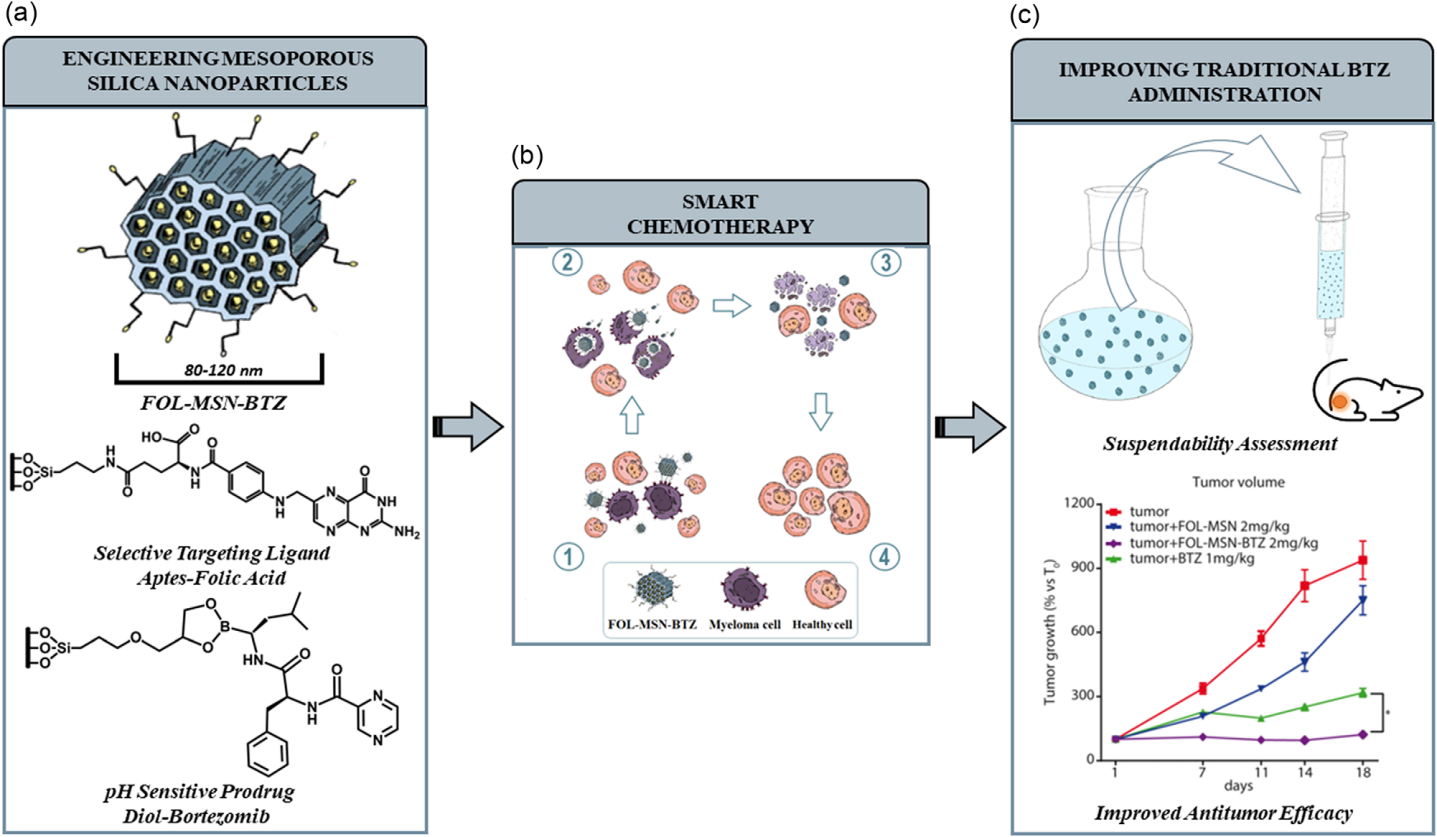
Graphical representation of FOL‐MSN‐BTZ development, mechanism of internalization and administration. a) FOL‐MSN‐BTZ engineering with evidence of functionalization structural details; b) mechanism of FOL‐MSN‐BTZ uptake in cancer cells: 1) interaction of the device through folic acid with folate receptors (FR) overexpressed in cancer cells; 2) internalization of FOL‐MSN‐BTZ only in cancer cells where bortezomib is released in response to tumor acidic microenvironment; 3) MM cell death; 4) healthy cells unaffected by FOL‐MSN‐BTZ administration; c) suspensions preparation, administration and in vivo performances. Reproduced with permission.^[^ ^25^ ^]^ 2022, The Royal Society of Chemistry.

Materials engineering is the emerging ability to nanostructure the matter. It makes possible the development of new materials that are themselves devices. The biological systems, in general, are a source of inspiration for designing the solution for solving problems in the fields of bionanotechnology, being the only limits the imaginative potential of the materials engineer or the details of the knowledge of the inspirating system itself.^[^ ^24^ ^]^ We have been active, until now, in the development of nanostructured devices for nanomedicine applications in the field of chemotherapies. This discussion will be mainly focused on mesoporous silica‐based nanostructured devices and targeted delivery strategies for approaching the development of the related nanodevices. Recently we have presented an MSU‐type mesoporous silica‐based nanodevice (FOL‐MSN‐BTZ), able to selectively deliver the antineoplastic drug bortezomib (BTZ) to folate receptor overexpressing multiple myeloma (FR + MM) cells.^[^ ^25^ ^]^ The receptor‐specific ligand, folic acid, grafted on the external surface of the nanosystem, allows tumor recognition and cell internalization, while BTZ, mainly linked to the pore internal surface through a covalent pH‐sensitive bond, is released specifically in the acidic environment of the tumor (**Figure** **4**). A detailed investigation revealed that only the fine balancing of different functionalities on both the external and internal surfaces of MSNs ensures the absence of toxicity toward healthy cells in vitro and negligible BTZ release at physiological pH.

These features make the nanodevice well‐suited for applicative purposes in the engineering of therapies. This represents a process that involves both the development of a nanodevice designed to manage drug release in therapeutically relevant areas within the body (Figure 4a,b) and the preparation of the nanodevice in a form (suspension in this case, Figure 4c) that can be efficiently administered in the organism.

After comprehensive in vitro characterization, an accurate evaluation of suspendability (Figure 4c) was conducted, considering the sedimentation process that decreases particle amount and, consequently, the drug content in the injected suspension volume. This assessment enabled the development of an injectable formulation of FOL‐MSN‐BTZ that showed higher antitumor efficacy and demonstrated an overall tendency toward lower toxicity in a MM mice model compared to conventional bortezomib chemotherapy (Figure 4c).

In our experience we have observed that excessive external functionalization can block the movement of nanoelements through the pores that are essential for the nanostructuring processes. Conversely, a reduced concentration of receptor‐specific ligand may hinder the interaction between the nanodevice and the biological structures.

Surface engineering has also emerged as a crucial design parameter for the in vivo combined (theranostic agent) applications of nanoparticles.^[^ ^26^ ^]^ In a recent study, Farjadian and coworkers described a multifunctional pH‐responsive nanoplatform designed for efficient drug delivery and magnetic resonance imaging. The development of the nanoparticles has been carried out according to a multistep procedure. They employed engineered hyaluronan‐coated ethylenediaminetetraacetic (EDTA)‐modified magnetic mesoporous silica nanoparticles for cisplatin drug delivery. Hyaluronic acid (HA) was used as target‐specific CD44 ligand while EDTA, that is able to complex cisplatin and release it in a pH‐responsive way, was incorporated for platinum‐based drug loading. The resulting nanosystem showed an excellent efficacy for delivering the drug to CD44 receptor overexpressed tumor cells, exhibiting a high adsorption capacity of cisplatin that was gradually released in the acidic environment. Additionally, the biocompatibility and biodegradability of the EDTA‐MSN@HA vehicle were confirmed.

Recently, mesoporous silica nanoparticles for MiR‐200c‐3p delivery for breast cancer treatment have been presented. miR‐200c‐3p is a well‐known tumor suppressor microRNA that inhibits tumor progression and metastasis in breast cancer through downregulating ZEB1 and ZEB2. The final nanodevice, prepared by following a layer‐by‐layer procedure on MSNs, contains a core of MSN, a first layer of polyethylenimine (PEI) followed by layers of miR‐200c‐3p and HA (MSNPEI‐miR200c‐HA). PEI was attached to the surface of MSN by electrostatic interactions due to its positive charge (amine groups). HA and miR‐200c‐3p were bound to MSN‐PEI also electrostatically, due to their negative charge (carboxy group in the case of HA, and orthophosphoric group in the case of miR‐200c‐3p). Hyaluronic acid has been employed for the targeting of CD44 receptor.^[^ ^27^ ^]^

Zang et al.^[^ ^28^ ^]^ reported a visible light‐responsive biodegradable mesoporous silica‐based nanosystem, bMSNs‐AZO/CD‐PMPC, for both drug delivery and lubrication enhancement in osteoarthritis treatment. The synthesized system is based on host‐guest interaction between azobenzene (AZO) and beta‐cyclodextrin (CD) modified poly(2‐methacryloxyethylphosphorylcholine) (PMPC) grafted on the external surface of MSNs which could block the mesopores. The light‐responsive controlled release of the anti‐inflammatory drug diclofenac sodium (DS) was triggered by 450 nm visible light, inducing the partial *trans*‐to‐cis isomerization of AZO and thus dissociation of CD‐PMPC from nanoparticle surface. Additionally, the hydrated layer surrounding the zwitterionic charged groups (N^+^(CH_3_)_3_ and PO_4_^−^) in CD‐PMPC improved lubrification, resulting in a remarkable reduction of friction coefficient (COF) value, beneficial for treatment of osteoarthritis. In vitro studies demonstrated good cell compatibility and promising anti‐inflammatory properties of the nanodevice, providing for the first time the design of a stimuli‐responsive nanosystem using visible light irradiation for osteoarthritis therapy.

Shi et al.^[^ ^28^, ^29^ ^]^ have engineered a dual‐targeting multifunctional nanoplatform based on a pH‐sensitive mesoporous calcium silicate nanocomposite (MSNs) designed to selectively target the tumor microenvironment, providing an effective combination of photothermal therapy (PTT) and photodynamic therapy (PDT) for treating breast cancer. Calcium ions were introduced during the synthesis of nanocomposites to confer biodegradability to the mesoporous silica carriers. In tumor microenvironment, macrophages promote cancer initiation and malignant progression. Tumor‐associated macrophages (TAMs) are predominantly polarized into the “alternatively activated” M2‐like macrophages.

M2‐like TAMs promote tumor growth and progression by producing stromal catabolic factors and mediating immunosuppression. For this reason, they are considered an attractive targets for improving the efficacy of cancer treatment.

The mannosylated hyaluronic acid (Man‐HA) surface modification through alendronate moiety (ALE) allowed tumor tissue enrichment of nanoparticles not only by EPR effect, but also by simultaneously targeting both CD44 and CD206 receptors highly expressed in breast cancer cells and in M2‐like tumor‐associated macrophages (TAMs), respectively. The encapsulated indocyanine green (ICG) exposed to 808 nm NIR light, generated local heat triggering production of ROS and consequently improving the anti‐tumor efficacy of PTT/PDT. The addiction of calcium ions in silica farmwork conferred pH‐dependent rapid decomposition of silica backbone and biodegradation of inorganic carrier. The nanovehicle was inert under physiological conditions but was disrupted in acidic environments, promoting cell apoptosis in vitro and in vivo investigations.

De Cola et al. introduced an alternative strategy to finely regulate the degradation of MSN employing a stimuli‐responsive mechanism.

They incorporated imine groups into the silica framework for this purpose. Particles with different contents of imine groups have been investigated to assess their impact on the physicochemical properties. The degradation rate was found to be pH‐dependent, with fast degradation observed at slightly acidic conditions. This is noteworthy since silica particles typically exhibit high stability in acidic media.^[^ ^30^ ^]^

Our research group has developed a targeted mesoporous silica‐based drug delivery device designed for bone‐specific drug delivery. Specifically, alendronate, a prominent member of the diphosphonate drug class, has been electrostatically bonded to the external carboxyl groups of a suitable modified mesoporous silica. Alendronate served as a targeting function for bone tissue, while ibuprofen was used as the model drug delivery. To investigate the system's performance, we conducted preliminary studies involving the synthesis and application of hydroxyapatite, an inorganic phase mimicking the bone matrix. These experiments aimed to assess the interaction between the ligand alendronate and the hydroxyapatite.^[^ ^31^ ^]^

Chen and colleagues synthesized a uniform and size‐controllable hollow mesoporous silica nanosystem (HMSN).^[^ ^32^ ^]^ This study highlights in vivo tumor‐targeted dual‐modality imaging, leveraging positron emission tomography (PET) and near‐infrared fluorescence (NIRF), and an enhanced drug delivery of HMSN through a widely applicable surface engineering technique. This achievement was realized through a modified hard‐templating method and a surface engineering process that involved functionalization with amino groups. The comprehensive modification process included loading the anticancer drug doxorubicin (DOX) into the hollow space, incorporating near‐infrared dye (ZW800) and copper chelator (NOTA) linkages, PEGylation with SCM‐PEG5k‐Mal, conjugation with the antibody TRC105, and radiolabeling with the PET isotope 64Cu. In‐depth in vitro and in vivo studies were conducted to assess various aspects of the HMSN nanoconjugate, including stability, tumor targeting efficacy and specificity, biodistribution, and drug delivery capability. The results revealed enhanced tumor uptake in a 4T1 murine breast cancer model, highlighting the specific targeting behavior of TRC105 toward CD105 on tumor neovasculature. Additionally, the hollow space inside the nanovehicle was found to improve the loading capacity of DOX compared to conventional mesoporous silica nanoparticles (MSN).

Nanodynamic therapies, including the above‐cited PTT and PDT, have increasingly gained attention as treatment strategies for deep‐seated tumors by inducing the generation of free radicals or reactive oxygen species (ROS) directly within the tumor site. Activation of nanosensitizers can occur through either exogenous or endogenous triggers, such as internal chemical or biological reactions. MSNs or MSNs‐based hybrid nanoparticles show interesting potentialities for application in nanodynamic therapies. A recent review by Zhao et al. examines the antitumor mechanism, challenges, and development prospects of MSN‐based nanodynamic therapies and multi‐dynamic combination therapies for clinical applications.^[^ ^33^ ^]^

**Figure 5:**
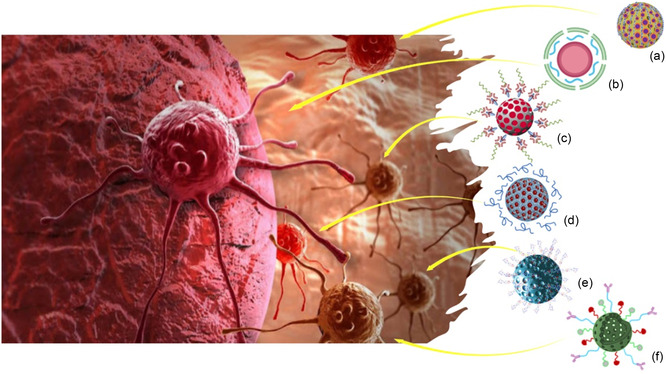
Examples of advanced surface engineered nanoplatforms for nanomedicine applications. a) Hyaluronan‐coated EDTA‐modified magnetic MSN for targeted cisplatin delivery and MIR imaging;^[^ ^26^ ^]^ b) PEI and hyaluronic acid modified MSN for targeted MiR‐200c‐3p delivery in breast cancer therapy;^[^ ^27^ ^]^ c) light‐responsive dual functional biodegradable MSN grafted with azobenzene and beta‐cyclodextrin‐modified PMPC for controlled diclofenac sodium release;^[^ ^28^ ^]^ d) modified alendronate‐mannosylated hyaluronic acid mesoporous calcium degradable nanocomposite for combined photothermal and photodynamic therapy in breast treatment;^[^ ^29^ ^]^ e) mesoporous silica‐based nanodevice, grafted with alendronate for bone‐specific delivery of ibuprofen;^[^ ^31^ ^]^ f) aminopropyl functionalized HMSN, modified with near‐infrared ZW800 dye, NOTA chelator, thiolated anti‐CD105 antibody for imaging and treatment of breast cancer.^[^ ^32^ ^]^ Reproduced under terms of CC‐BY license.

In **Figure** **5** many of the earlier discussed MSN‐based nanodevices for nanomedicine applications are schematically represented.

A comprehensive presentation of the targeted delivery strategies based on MSN is out of the aim of this perspective paper. Nevertheless, we have summarized the features of the presented and discussed strategies and solutions in **Table** **1** to give an immediate representation of the explored nanostructuring potentialities with the MSN.

**Table 1:** Details of the discussed targeted delivery strategies based on MSN.

References	Active targeting	External receptor‐specific ligand	Stimuli‐responsivity	Diagnostic	Framework degradation	Dual drug release
[25]	✓	✓	✓	–	–	–
[26]	✓	✓	✓	✓	–	–
[27]	✓	✓	✓	–	–	–
[28]	–	–	✓	–	–	✓
[29]	✓	✓	✓	–	✓	–
[30]	–	–	✓	–	✓	–
[31]	✓	–	–	–	–	✓
[32]	✓	✓	✓	✓	–	–

### 3.1 Potentialities and Challenges of Nanomedicine

The evolution of nanotechnology has significantly revolutionized several scientific areas. This emerging technology has found extensive applications in pharmaceutical and medical research. The application of nanotechnology for medical purposes, referred as nanomedicine, involves the utilization of nanoscale materials for disease diagnosis, monitoring, control, prevention, and treatment.^[^ ^34^ ^]^ A key strength of nanomedicine lies in its ability to overcome limitations associated with traditional active substances and conventional drug administration methods, thereby addressing unmet medical needs.^[^ ^35^ ^]^ By employing nanomedicine formulations, active pharmaceutical agents achieve enhanced solubility and stability, and are selectively delivered to target sites without affecting healthy tissues. This leads to an improved pharmacokinetic profile, increased drug bioavailability, reduced dosage requirements, enhanced efficacy, and safety, and minimized adverse effects.^[^ ^36^ ^]^

Doxil, the first FDA‐approved nanodrug, was introduced in 1995 for the treatment of specific cancers, including metastatic ovarian cancer and AIDS‐related Kaposi's sarcoma.^[^ ^37^ ^]^

According to 2021 data, there are currently 100 nanomedicines available in the market, with an additional 563 undergoing clinical trials or at different developmental stages, totaling 663.^[^ ^38^ ^]^ An important step forward in the nanomedicine field concerns the development of a bottlebrush prodrug (BPD) platform designed specifically for multiple myeloma therapy. This platform incorporates a combination of anti‐multiple myeloma (MM) drugs: bortezomib, a proteasome inhibitor, pomalidomide, an immunomodulating agent, and dexamethasone, a corticosteroid. BPDs carrying a statistical mixture of these three drugs in a synergistic ratio, exhibit superior performance compared to both the free‐drug combination at the same ratio and a mixture of single‐drug BPDs in the identical ratio. The enhanced therapeutic outcomes are attributed to the precise combination of these drugs and their co‐formulation within a nanoparticle delivery system, allowing for the use of significantly lower drug doses. This innovative strategy holds great promise for elevating the effectiveness of nanomedicine applications.^[^ ^39^, ^40^ ^]^

Nevertheless, the transition of nanomedicine from small‐scale laboratories to large‐scale industrial production and market availability remains a slow, challenging, and often unsuccessful process.

Regulatory guidelines mandate a comprehensive assessment of nanomaterial‐based drug products, including physico‐chemical attributes, stability, quality, and safety checks.^[^ ^41^ ^]^

Unfortunately, a significant hindrance to the clinical translation of nanomedicine is the lack of specifically implemented guidelines and protocols for evaluating cutting‐edge technologies.

Given the complex composition of several components for designing nano‐formulations, it is crucial to employ sophisticated and appropriate procedures to exhaustively characterize them, surpassing the requirements for traditional pharmaceuticals. In fact, the manufacturing process of an engineered nanodevice involves intricate steps such as chemical surface modification, conjugation of targeting ligands, and drug anchoring, necessitating continuous control over nanomaterial properties, including size, shape, morphology, charge,^[^ ^42^ ^]^ and biopharmaceutical behavior. Consequently, more rigorous characterizations are imperative to advance the development of nanomedicine. Another crucial issue associated with nanomedicine is the safety risk inherent in delivering nanomaterials into the human body. Assessing their long‐term toxicity involves assays that are currently non‐standardized and inadequate, as they were originally designed for traditional drugs.^[^ ^43^ ^]^

The current lack of a clear and definite understanding of the in vivo biological effects of nanosystems is a major obstacle in bridging the gap between laboratory research and clinical application. In light of these challenges, the development of nanomedicine becomes an urgent need, demanding a meticulous investigation of chemistry and manufacturing processes, comprehensive characterizations, bioperformance assessments, safety considerations, and adherence to regulatory affairs.

## 4 Molecular Targeted Therapies

Unlike cytotoxic targeted therapies, MTT exert their anticancer effects through various mechanisms, including the inhibition of cell proliferation, metastasis, and angiogenesis. Additionally, these therapies can induce apoptosis and reverse multidrug resistance. Recent advancements in molecular profiling of cancer have led to the approval of more and more safe and effective targeted therapeutic agents, thereby providing novel treatment opportunities.^[^ ^44^ ^]^

Tumor‐pecific gene mutations, absent in normal cells, drive the production of aberrant proteins that, when identified, serve as targets for the development of targeted therapies. These therapies are designed to specifically act on the abnormal proteins encoded by mutated genes. Over the last two decades, drug discovery strategies have shifted their focus toward identifying tumor‐specific druggable mutations and developing corresponding targeted agents.

The progressive adoption of this approach for selecting appropriate targeted therapies has been made possible by recent advancements in sequencing technologies, particularly in next‐generation sequencing (NGS). NGS concerns “…the colloquial way to describe highly parallel of high‐output sequencing methods that produce data at or beyond the genome scale”.^[^ ^45^ ^]^

**Figure 6:**
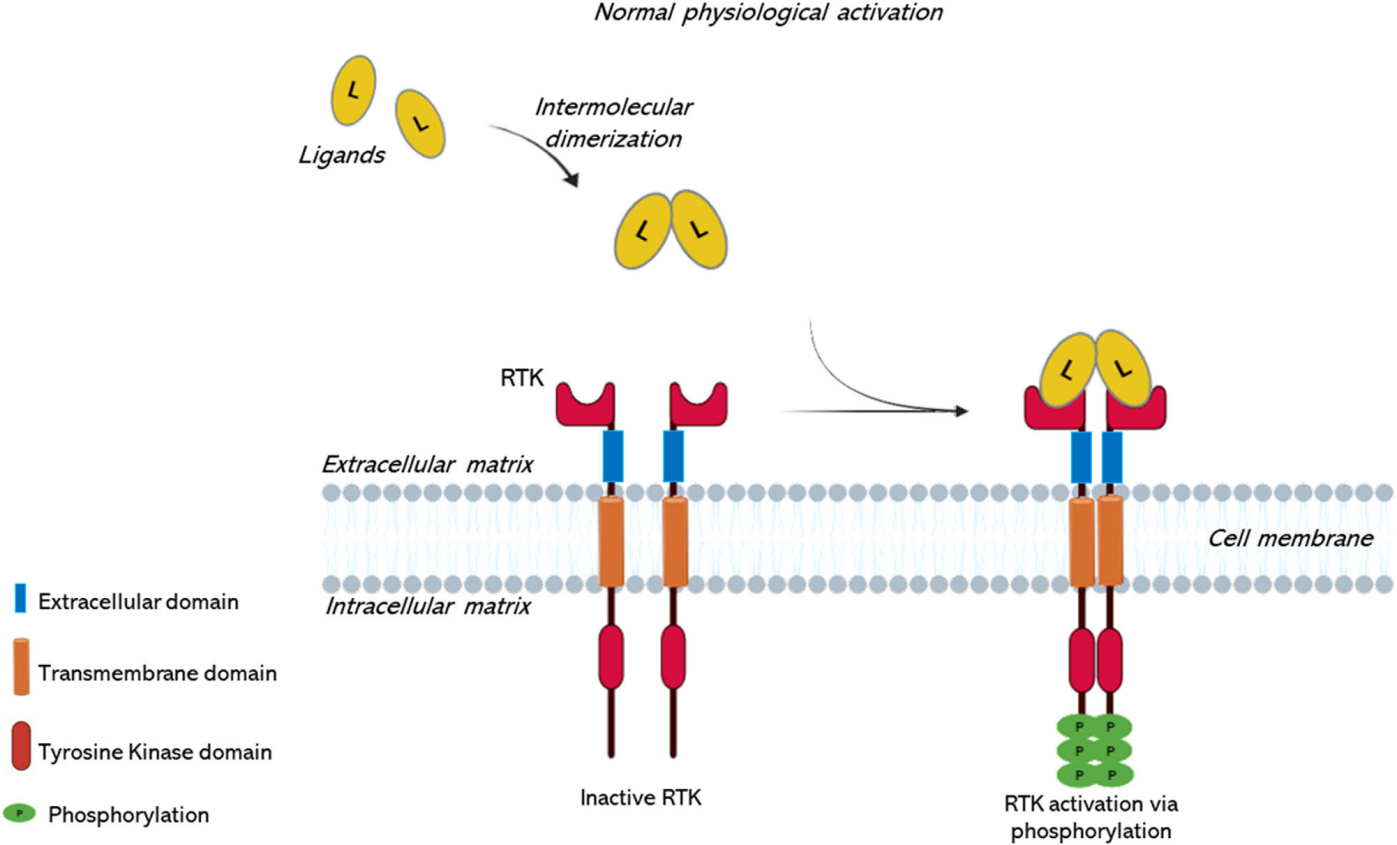
Activation mechanism of RTK. Schematic representation illustrating the typical process of healthy RTKs through ligand‐induced intermolecular dimerization, leading to activation of the intracellular TKD under physiologically normal conditions, thereby initiating downstream signaling cascades.

Protein kinases constitute a family of enzymes, involved in signaling pathways, and responsible for phosphorylating target proteins by transferring a phosphate group from ATP to specific amino acid residues containing hydroxyl groups. Phosphorylation, the most common protein modification, plays a crucial role in signal transmission. Under normal physiological conditions, receptor tyrosine kinases (RTKs), are typically activated by receptor‐specific growth‐factor ligands that bind to the extracellular regions of RTKs, thereby inducing receptor dimerization and/or oligomerization (**Figure** **6**).^[^ ^46^, ^47^ ^]^

For most RTKs, the intracellular inactive tyrosine kinase domain (TKD) is characterized by a *cis*‐autoinhibition state resulting from a set of intramolecular interactions that vary from receptor to receptor. The diverse regulatory mechanisms are contingent upon the distinct structures of inactive TKDs. The ligand‐induced dimerization of the extracellular regions of RTKs triggers the activation of the intracellular TKD.

In the activated state, a specific configuration is achieved by all TKDs, which is essential for catalyzing phosphotransfer.^[^ ^48^ ^]^ This conformational change enables *trans*‐autophosphorylation of each TKD, resulting in the release of *cis*‐autoinhibition, and subsequent recruitment and activation of a diverse array of downstream signaling proteins.

**Figure 7:**
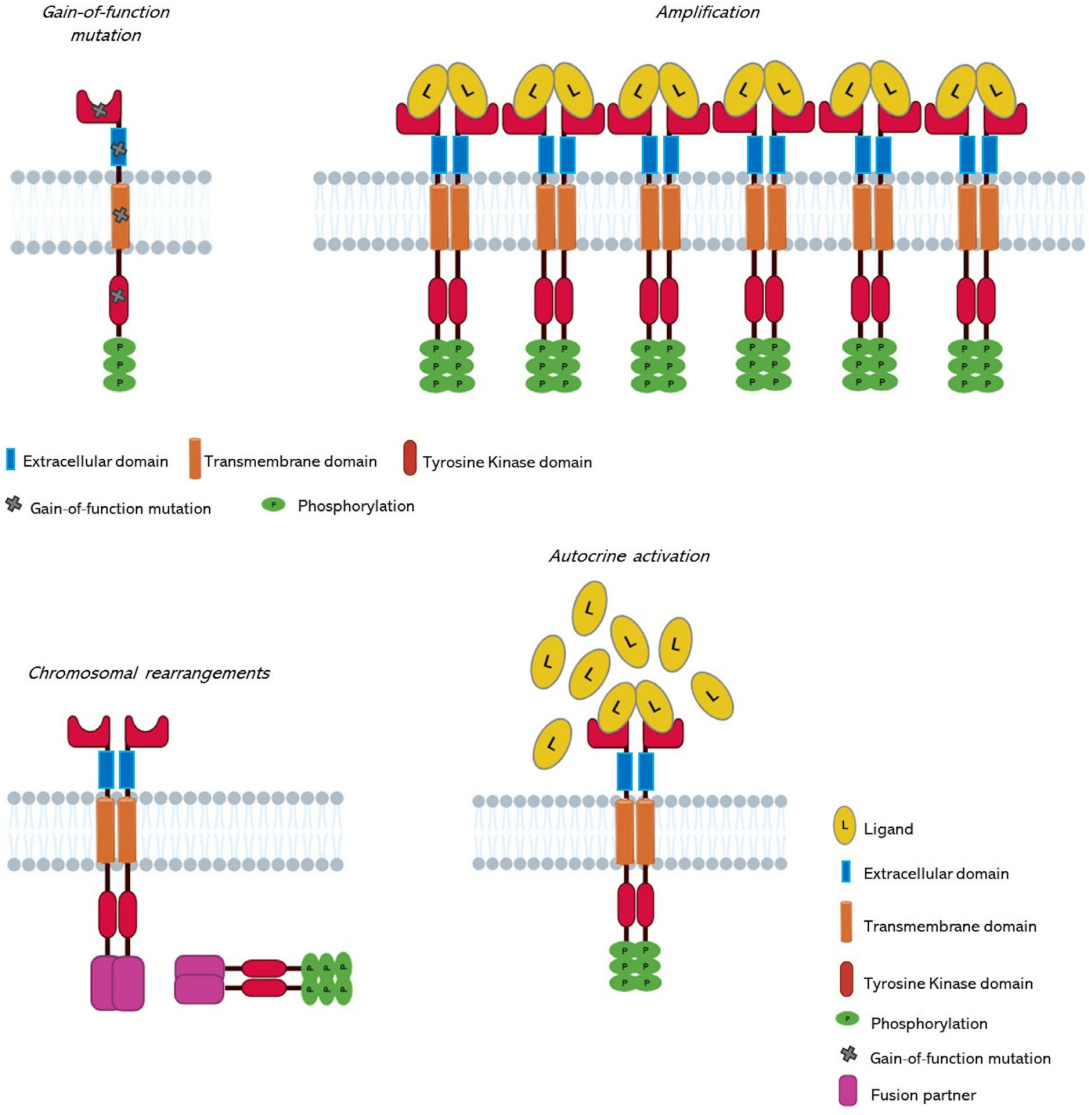
Different mechanisms of RTK‐induced oncogenesis: 1) Gain‐of‐function mutations, inducing ligand‐independent kinase activation without receptor dimerization; 2) Overexpression of RTKs, enhancing surface receptor concentration, promoting receptor dimerization, and subsequent kinase activation; 3) Chromosomal rearrangements, forming hybrid fusion oncoproteins comprising RTK and fusion partner components, resulting in activated kinase domains; 4) Autocrine activation, wherein cancer cell‐produced ligands activate the same cells. The increased local ligand concentration induces RTK activation, resulting in RTK dimerization, enhanced kinase activity, and receptor C‐terminal tail phosphorylation.

Moreover, various aberrant mechanisms have been described, encompassing acquired mutations in RTK genes, such as gain of function mutations, duplications or signaling amplification, rearrangements, or alternative mechanisms that induce receptor activation via autocrine pathways, as illustrated in **Figure** **7**.

This oncogenic activation disrupts the balance between cell growth/proliferation and cell death.^[^ ^49^ ^]^

Blocking the signaling pathways mediated by dysregulated Protein Kinases Receptors can reduce the onset and progression of various cancer types.

Among protein kinase receptors, mutated RTKs offer a wealth of potential targets, made accessible by advancements in sequencing. Simultaneously, the progress in chemical approaches has made many of these targets suitable for drug development.

Tyrosine kinases, mainly act as receptors for growth factors or interact directly with these receptors, while serine/threonine kinases, respond to various cellular cues, including signals downstream of tyrosine kinases.^[^ ^50^ ^]^ Kinase inhibitors exert their effects by competing with ATP within the ATP‐binding pocket of the kinase, interacting with either the active (type I) or inactive (type II) conformation. Additionally, kinase inhibitors may function through allosteric mechanisms (types III and IV) by binding to regions other than the ATP‐binding pocket of the kinase, and/or other mechanisms.^[^ ^51^, ^52^ ^]^ Apart from chemical inhibitors, monoclonal antibodies (mAbs) present a promising approach for targeting tyrosine kinases extracellular domains.^[^ ^53^, ^54^ ^]^

Tyrosine kinases play a pivotal role in conveying signals from extracellular ligands to downstream effectors in cellular signaling pathways. These effectors predominantly include serine/threonine kinases, along with other crucial proteins like RAS, and they receive signals from ligands to extracellular tyrosine kinases. Mutations in the latter can induce oncogenesis by activating downstream signaling pathways. Additionally, mutations in serine/threonine kinases are common in cancers and can be targeted similarly to tyrosine kinases. However, monoclonal antibodies (mAb) cannot be utilized for serine/threonine kinases due to the absence of an extracellular domain.^[^ ^50^ ^]^

### 4.1 Dual Targeting Inhibitors

The search for more effective therapeutic interventions has led to continuous exploration of innovative approaches in drug discovery, particularly in response to the limited efficacy of single‐target drugs. Among these, the design of multitarget compounds has gained importance, with chimeric molecules representing a significant advancement in this field. A chimeric molecule is a nanostructured entity formed by linking two or more components to create a novel agent with distinct biological activities.^[^ ^55^ ^]^

Bifunctional single drugs, which combine two inhibitors targeting specific biomolecules, have emerged as promising alternatives. This approach has demonstrated enhanced antitumor effects and reduced toxicities.^[^ ^56^ ^]^ The development of dual inhibitors involves combining two starting molecules into a single hybrid compound. These dual inhibitors exhibit increased activity, reduced toxicity, and improved therapeutic index and bioavailability.^[^ ^57^ ^]^

Histone deacetylases (HDAC) have been widely investigated as novel potential anticancer drug targets, given their involvement in critical biological processes such as cell‐proliferation, metastasis, and apoptosis. The limited efficacy and selectivity of HDAC inhibitors can be overcome by developing them as part of dual‐targeting inhibitors.^[^ ^58^ ^]^

### 4.2 PROTAC, ADC and Bispecific Antibodies Binding to Two Different Targets

**Figure 8:**
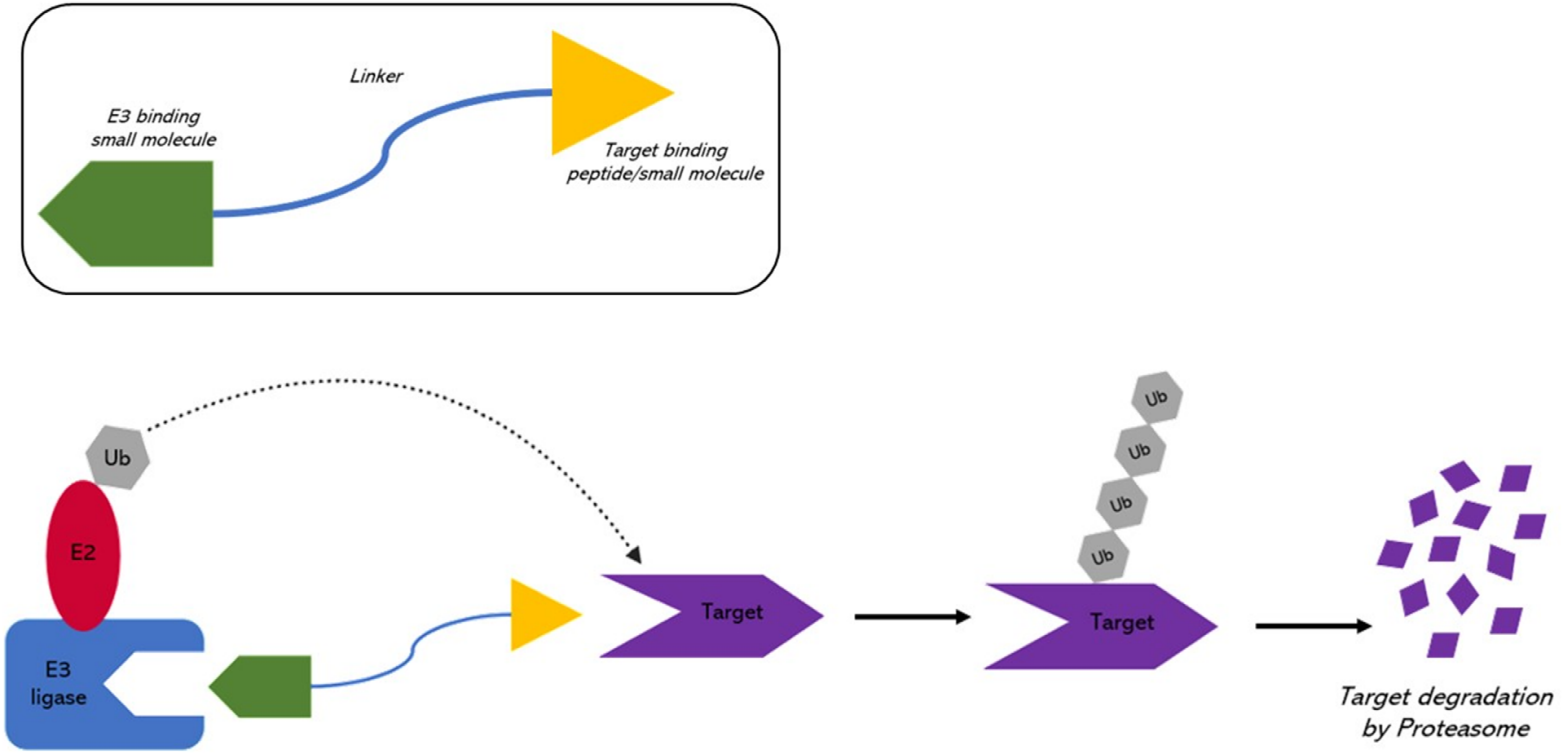
Mechanism of target protein degradation induced by PROTAC. PROTAC involves the linkage of two ligands via a linker: one ligand recruits E3, while the other binds to the protein of interest (POI). By simultaneously binding to both the target protein and the E3 ligase, PROTAC facilitates the formation of a ternary complex. Subsequently, the target protein undergoes polyubiquitination, marking it for degradation by the proteasome.

In the landscape of cancer‐targeted therapies, the proteolysis targeting chimera strategy (PROTAC, **Figure** **8**), a technology for targeted protein degradation, emerges as an innovative methodology. Diverging from traditional “target occupying” inhibitor therapies,^[^ ^59^ ^]^ PROTAC reduces the activity of target proteins by inducing their degradation. This strategy employs a chimera molecule,^[^ ^55^ ^]^ able of targeting and degrading the proteins of interest (POI). PROTAC molecule consists of a small molecule that binds to the targeted protein and a ligand of an E3 ubiquitin ligase (E3). This dual functionality brings the target protein and the ubiquitylation moiety into close proximity leading to the subsequent degradation of the POI by the ubiquitin−proteasome system (UPS).^[^ ^60^ ^]^

Various proteins, including estrogen‐related receptor alpha (ERRα), the serine‐threonine kinase RIPK2, and those featuring the bromodomain and extra‐terminal motif (BET),^[^ ^61^, ^62^, ^63^ ^]^ have become targets of interest.

PROTAC, recognized as a promising approach for targeted therapy drug development,^[^ ^64^ ^]^ has demonstrated efficacy in the targeted protein degradation of BCR‐ABL1 in chronic myeloid leukemia, emphasizing the potential benefits of concurrent inhibition and degradation of BCR‐ABL.^[^ ^65^ ^]^

PROTAC compounds have been employed to degrade the mitotic kinase AURORA‐A, a crucial cancer target. This was achieved by linking a clinical kinase inhibitor of Aurora‐A to E3‐ligase binding molecules.^[^ ^66^ ^]^

Two PROTAC drugs, ARV110 and ARV‐471, designed for treating prostate cancer and breast cancer, respectively, have progressed to phase II clinical trials.^[^ ^59^ ^]^ The year 2022 marked an increase in the number of PROTACs entering clinical trials,^[^ ^67^ ^]^ showing the growing exploration and interest in this innovative approach within the field of targeted cancer therapy.

**Figure 9:**
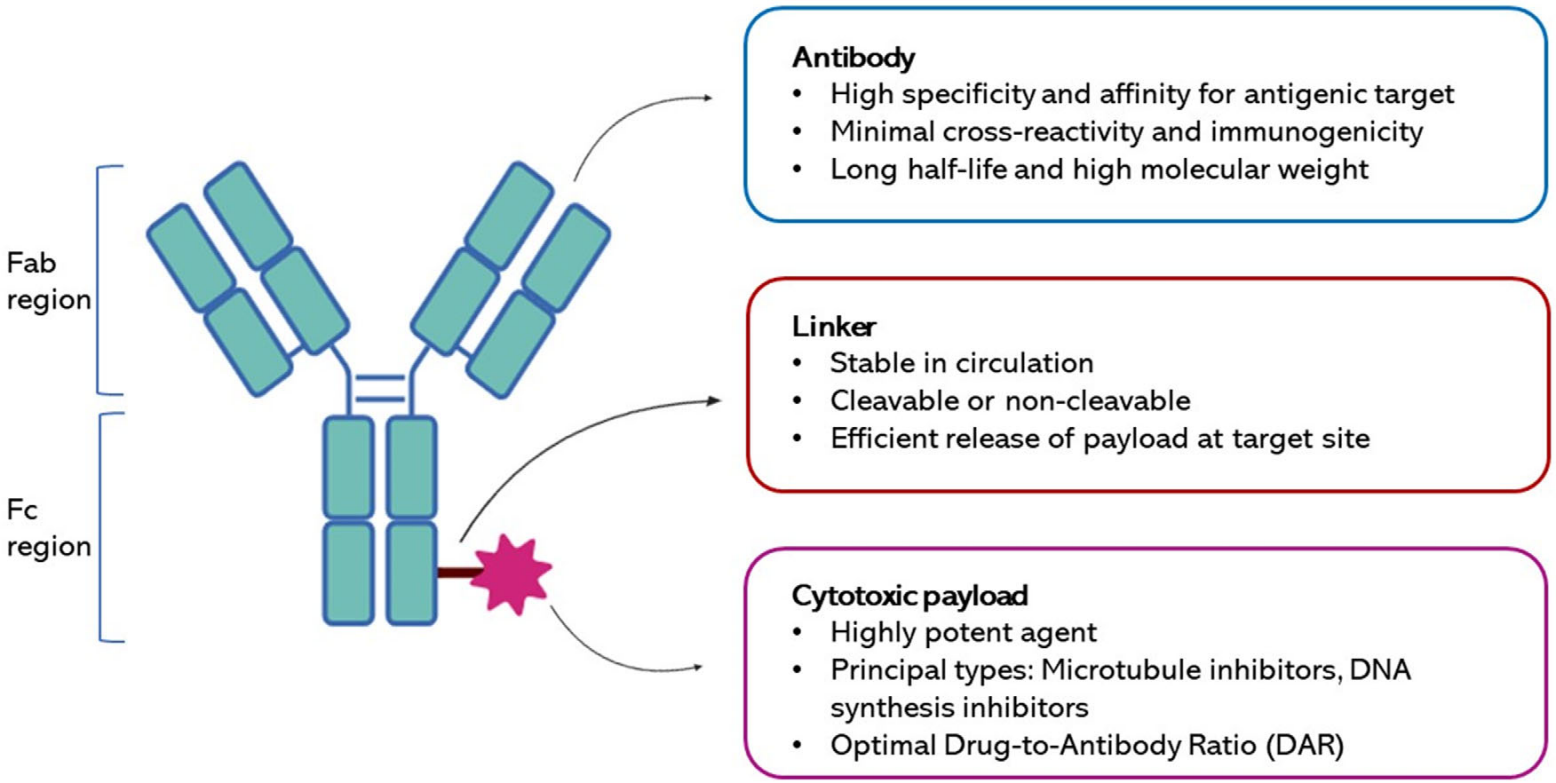
Schematic representation of antibody‐drug conjugates (ADC) approach for cancer therapy. An ADC comprises three key components: 1) the antibody (Ab) targeting a specific cell; 2) the linker; 3) the cytotoxic drug. They work synergistically to enable precise delivery of the cytotoxic agent to the target cell. ADCs are engineered to stay stable in the bloodstream and release the drug inside cells after binding to a specific antigen. The antibody needs to exhibit high binding affinity to the specific target antigen, low immunogenicity, stability in the bloodstream, and minimal cross‐reactivity. The linker connects the antibody to the cytotoxic payload via covalent conjugation, ensuring stability in the bloodstream and enabling quick release of the drug at the target site. Successful ADC development also relies on the drug‐antibody ratio (DAR), which quantifies the number of drug molecules attached to the antibody via the linker.

Employing antibodies as targeting agents presents a strategic approach for delivering cytotoxic payloads to specific tissues. In this approach, a tumor‐specific antibody is connected to a cytotoxic drug through a linker (**Figure** **9**).^[^ ^68^ ^]^

Notable examples of this strategy include HER2‐targeted antibody‐drug conjugates like trastuzumab‐emtansine, trastuzumab‐deruxtecan, and trastuzumab‐duocarmazine.^[^ ^69^, ^70^ ^]^ The targeting selectivity of antibodies joined to the drug loading ability of nanoparticles seems to disclose potential advancement in the field.^[^ ^71^ ^]^

Another innovative approach involves bivalent monoclonal antibodies (mAbs) able of targeting two oncogenic drivers simultaneously. Recently, amivantamab, a bivalent mAb targeting epidermal growth factor receptor (EGFR) and mesenchymal‐epithelial transition factor (MET), received approval for the treatment of non‐small cell lung cancer (NSCLC).^[^ ^72^ ^]^

Biparatopic bispecific antibodies (bsAbs) are designed to bind to two non‐overlapping epitopes on the same target.^[^ ^73^ ^]^ Zanidatamab, a humanized bispecific monoclonal antibody, is directed against two non‐overlapping domains of HER2.^[^ ^74^ ^]^

Although a single Zanidatamab molecule may not bridge the distance between the two antigen‐binding domains, it binds HER2‐expressing tumor cells with increased Ab saturation compared to canonical monospecific antibodies.^[^ ^75^ ^]^

## 5 Multi‐Targeting Inhibitors

Certain drugs exhibit limited efficacy due to their single‐targeting nature, for this reason polypharmacology can represent the next paradigm in drug discovery.^[^ ^76^ ^]^

Combined therapies univocally show advantages over monotherapies. **Table** **2** presents the statistical analysis results of the discussed drug combinations that have been translated into clinical trials and compared with the corresponding monotherapies or dual‐target therapy versus placebo. It has been observed that multi‐targeted molecular therapy exhibits a superior median progression‐free survival compared to single‐targeted therapy.

**Table 2:** Statistical analysis results of combination therapies and related monotherapies.

Clinical Trial (NCT*) and Study Phasea)	Subset Cancer Patient	Therapy	Clinical Endpoint and Results	Study Description	Reference
PAPILLON (NTC04538664), Phase III	Patients with NSCLC and EGFR exon 20 insertions in First Line	Amivantamab—Chemotherapy	Median PFS 11.4 months (95% C.I. 9.8 to 13.7);	Efficacy of use Amivantamab‐Chemotherapy combined as compared with chemotherapy alone	[113]
			ORR was 73% (95% C.I. 65‐80)		
		Chemotherapy	Median PFS 6.7 months (95% C.I. 5.6 to 7.3);		
			ORR was 47% (95% C.I. 39–56);		
MARIPOSA‐2 (NTC04988295), Phase III	Patients with NSCLC EGFR mutated (exon 19 deletions or L858R) locally advanced or metastatic after disease progression on Osimertinib	Amivantamab‐Lazertinib‐ Chemotherapy	Median PFS 8.3 months (95% C.I. 6.8 to 9.1);	Patients were Randomized 2:2:1 to receive Amivantamab‐Lazertinib‐ Chemotherapy, Chemotherapy‐ or Amivantamab‐ Chemotherapy	[114]
		Amivantamab‐ Chemotherapy	Median PFS 6.3 months (95% C.I. 5.6–8.4);		
		Chemotherapy	Median PFS 4.2 months (95% C.I. 4.0–4.4);		
KRYSTAL‐1 (NTC03785249), Phase I/II	Patients with advanced solid tumors harboring a KRAS p.G12C mutation.	Adagrasib monotherapy	Median PFS 5.6 months (95% C.I. 4.1–8.3);	Patient in non‐randomized clinical trial receive Adagrasib monotherapy or Adagrasib in combination with intravenous Cetuximab.	[115]
			Median OS 19.8 (95% C.I. 12.5–23.0)		
		Adagrasib plus Cetuximab	Median PFS 6.9 months; (95% C.I. 5.4–8.1);		
			Median OS 13.4 (95% C.I. 9.5–20.1)		
CodeBreaK 300 (NCT05198934), Phase III	Patients with chemorefractory metastatic Colorectal Cancer with mutated KRAS p. G12C	Sotorasib Panitumumab high dosage	Median PFS 5.6 months; (95% C.I. 4.2–6.3); Objective response 26.4% (95% C.I. 15.3 to 40.3)	Study multicenter, open label clinical trial that treated patients 960 mg Sotorasib Panitumumab or 240 mg Sotorasib Panitumumab or trifluridine‐tipiracil or regorafenib	[116]
		Sotorasib Panitumumab low dosage	Median PFS 3.9 months; (95% C.I. 3.7–5.8); Objective response 5.7% (95% C.I. 1.2–15.7)		
		Standard care: trifluridine‐tipiracil or regorafenib	Median PFS 2.2 months; (95% C.I. 1.9–3.9); Objective response 0% (95% C.I. 0–6.6)		
COSMIC‐313 (NCT03937219), Phase III	Patients with advanced renal‐cell carcinoma	Cabozantinib in addition to Nivolumab and Ipilimumab (experimental group)	Median PFS NR (14.0–NE);	Double‐blind trial that patients were randomly assigned toreceive 40 mg of Cabozantinib daily in addition to Nivolumab and Ipilimumab (experimental group) or matched placebo in addition to Nivolumab and Ipilimumab (control group)	[83]
			Objective response 43% (95% C.I. 37 to 49)		
		Placebo in addition to Nivolumab and Ipilimumab (control group)	Median PFS 11.3 months (7.7–18.2);		
			Objective response 36% (95% C.I. 30–42)		
CheckMate 9ER (NTC03141177), Phase III	Patients with untreated advanced renal‐cell carcinoma	Nivolumab plus Cabozantinib	median PFS 16.6 months; (95% C.I. 12.5–24.9); Objective response 55.7% (95% C.I. 50.1–61.2)	Randomized, open‐label trial, that patients received either nivolumab (240 mg every 2 weeks) plus cabozantinib (40 mg once daily) or sunitinib (50 mg once daily for 4 weeks of each 6‐week cycle)	[117]
		Sunitinib	Median PFS 8.3 months; (95% C.I. 7–9.7); Objective response 27.1% (95% C.I. 22.4–32.3)		

a) Non small cell lung cancer (NSCLC); epidermal growth factor receptor (EGFR); confidence interval (C.I.); progression free survival (PFS); overall response rate (ORR); overall survival (OS); not estimated (NE); not reached (NR).

Cabozantinib, a tyrosine kinase inhibitor (TKI), effectively targets VEGF receptors (VEGFRs), MET, AXL, and other receptor tyrosine kinases involved in tumor development through angiogenesis, metastasis, and drug resistance. Notably, Cabozantinib demonstrates statistically significant enhancements in overall survival, progression‐free survival, and objective response rate in patients with clear cell renal cell carcinoma (RCC) who had previously undergone treatment with a VEGFR TKI.^[^ ^77^ ^]^

The simultaneous modulation of multiple targets produces a synergistic effect, that results in a lower effective dose as well as fewer side effects.^[^ ^78^ ^]^

Furthermore, it has been shown that partial inhibition of several targets can result in a more pronounced effect than the complete inhibition of a single target. This synergistic effect specifically impacts the disease pathway, minimizing potential side effects in healthy tissues where target synergy is lacking.^[^ ^79^ ^]^

The cellular/tissue localizations of the targets should be compatible with a suitable accomodation of the ligands of an efficacious multiple‐targeting drug.

The ability of a molecule to bind to multiple proteins within a disease pathway is referred to as “promiscuity”, and it constitutes a key feature for a molecule to be considered a polypharmacologic drug candidate.

Unfortunately, promiscuity is not always easily related to molecular features. For instance, lipophilicity and ionization state are good predictors of promiscuity but molecular weight has been observed experimentally to be contradictorily related to promiscuity although it seemed, that could be selected as a paradigm for its identification.^[^ ^78^ ^]^

In the context of drug design, specific considerations come into play for non‐cleavable linkers, including attachment points to pharmacophores, as well as the length and geometry of the linker. Conversely, when dealing with ligands conjugated through a cleavable linker, careful attention must be given to the site of cleavage. This is particularly crucial as physiological conditions should induce the controlled release of the linked agent.

Furthermore, designing a drug with targets in different cells or tissues introduces complex challenges concerning pharmacodynamics, pharmacokinetics, and toxicology. This complexity necessitates the consideration of various aspects in drug development, ensuring both the efficacy and safety of the multi‐targeting drug.^[^ ^78^, ^80^ ^]^

The identification of target combinations in designing a multi‐targeting ligand is relatively straightforward for closely related targets, such as aminergic receptors or kinases,^[^ ^80^, ^81^, ^82^ ^]^ but becomes more challenging for unrelated targets. In various fields where the demand for multitargeted agents is increasing due to resistance development, such as in the field of multikinase inhibitors, the discovery of multitarget activity often relies on serendipity, followed by subsequent optimization.

Multitarget compounds can be developed according to three distinct pharmacophore architectures, with or without a linking group.

In the first case, the linker can be stable or biodegradable.^[^ ^83^, ^84^ ^]^ When stable, careful consideration is needed for attachment points, linker length, and geometry.^[^ ^85^ ^]^ In the biodegradable case, the release of linked molecules depends on the chemistry of the linker, determining whether and where cleavage occurs.^[^ ^84^ ^]^

However, this may result in an increase in the size of the molecules, making it challenging to reach intracellular compartments. Alternatively, when two bioactive small molecules are joined without a linker, fused multi‐targeting ligands are obtained. These can be either cleavable or uncleavable, depending on the nature of the employed bond. Small molecule multitarget agents can also result from the merging of multiple ligands.

Designing a merged pharmacophore that satisfies the biological targets of interest and optimizing it into a potent multitarget compound represents a highly challenging task.

## 6 Concluding Remarks

The limitations of nanomedicine solutions for chemotherapies are not due to the still available margins of improvements of efficacy or reduction of toxicity through smart administration of cytotoxic agents. Instead, these limitations arise from the superior nature of the advanced targeting concept associated with MTT. Unlike approaches limited to the internalization in cancer cells or responsiveness to pH stimuli for drug release, MTT operates at a more fundamental level by interfering with the biochemical mechanisms that drive cancer proliferation.

Recognizing that the administration of a single drug, even a highly effective small molecule, is insufficient to combat cancer, especially in light of promising results in the field of multi‐targeting molecules from polypharmacology, our perspective leans toward precision medicine facilitated by nanotechnology.

While molecular recognition and pH responsivity^[^ ^25^ ^]^ remain viable approaches, the strategic move is toward molecular multi‐targeted therapy.

The authors believe that the future of oncologic therapeutics lies in joined solutions between MTT and immunological therapies. Engineered mechanisms will guide these therapies, with nanotechnology playing a central role in drug design. However, in the context of this specific article and its focus, we propose a polypharmacology approach to develop a purely molecular multitargeting therapy, devoid of immunological elements.

The multi‐targeting drug that we imagine is not developed according to the approaches mentioned earlier, but through a nanotechnology‐based methodology, resulting in a molecular multi‐targeting nanodevice. It is ideally developed in our field of expertise but alternative potentially valid nanotechnological approaches cannot be excluded.^[^ ^86^, ^87^, ^88^ ^]^

Although this nanostructured device is speculative and, to the authors’ knowledge, hasn't been developed yet, it is based on previously demonstrated technical solutions in our earlier paper.^[^ ^25^ ^]^ The selection of functions is inspired by an ongoing trial [NCT04487080], evaluating the effectiveness of the amivantamab and lazertinib combination.

Amivantamab is a novel bispecific antibody that targets the extracellular domains of both EGFR and MET, effectively inhibiting tumor growth driven by EGFR and MET receptors.

Amivantamab^[^ ^72^, ^89^, ^90^ ^]^ employs three distinct potential mechanisms of action: ligand blocking, receptor degradation within lysosomal compartments (following the internalization of both EGFR and MET receptors in tumor cells), and immune cell‐directed activities, such as antibody‐dependent cellular cytotoxicity (ADCC), and, antibody‐dependent cellular trogocytosis (ADCT).^[^ ^91^ ^]^

Lazertinib (YH25448) is a novel irreversible third‐generation TKI developed for the treatment of EGFR mutant non‐small cell lung cancer.^[^ ^92^, ^93^, ^94^ ^]^ Lazertinib targets key activating mutations in EGFR, specifically the Exon 19 deletion or the Exon 21 L858R substitution, along with addressing the resistance mutation EGFR T790M.^[^ ^95^ ^]^

Therefore, our hypothesis, which we believe is reliable, is that a device could replace this combination by administering an equivalent drug combination within the microenvironment.

In simpler terms, we hypothesize a mesoporous silica‐based nanodevice externally conjugated, via an uncleavable bond, to a peptide mimicking^[^ ^96^, ^97^, ^98^ ^]^ Amivantamab antibody. Simultaneously, two distinct small molecules would be connected to the internal silica pore surface using a pH‐ultrasensitive bond, which can be hydrolyzed at the slightly acid interstitial pH typical of the tumor microenvironment.^[^ ^99^ ^]^

The solutions applicable to cleavable linkers^[^ ^100^ ^]^ have been thoroughly examined and may include linking groups susceptible to acidic conditions (hydrazones, oximes, and thiomaleimides),^[^ ^101^, ^102^, ^103^, ^104^ ^]^ or gatekeepers operating based on the principle of redox‐responsive drug release or other kind of responsivities.^[^ ^105^, ^106^, ^107^, ^108^ ^]^

In this proposed design, the hypothesized role of the antibody‐mimicking peptide is to block the extracellular components of target proteins, while the small molecules inhibitors can more easily penetrate the cell and inhibit the activities of intracellular target proteins.^[^ ^61^ ^]^

**Scheme 1:**
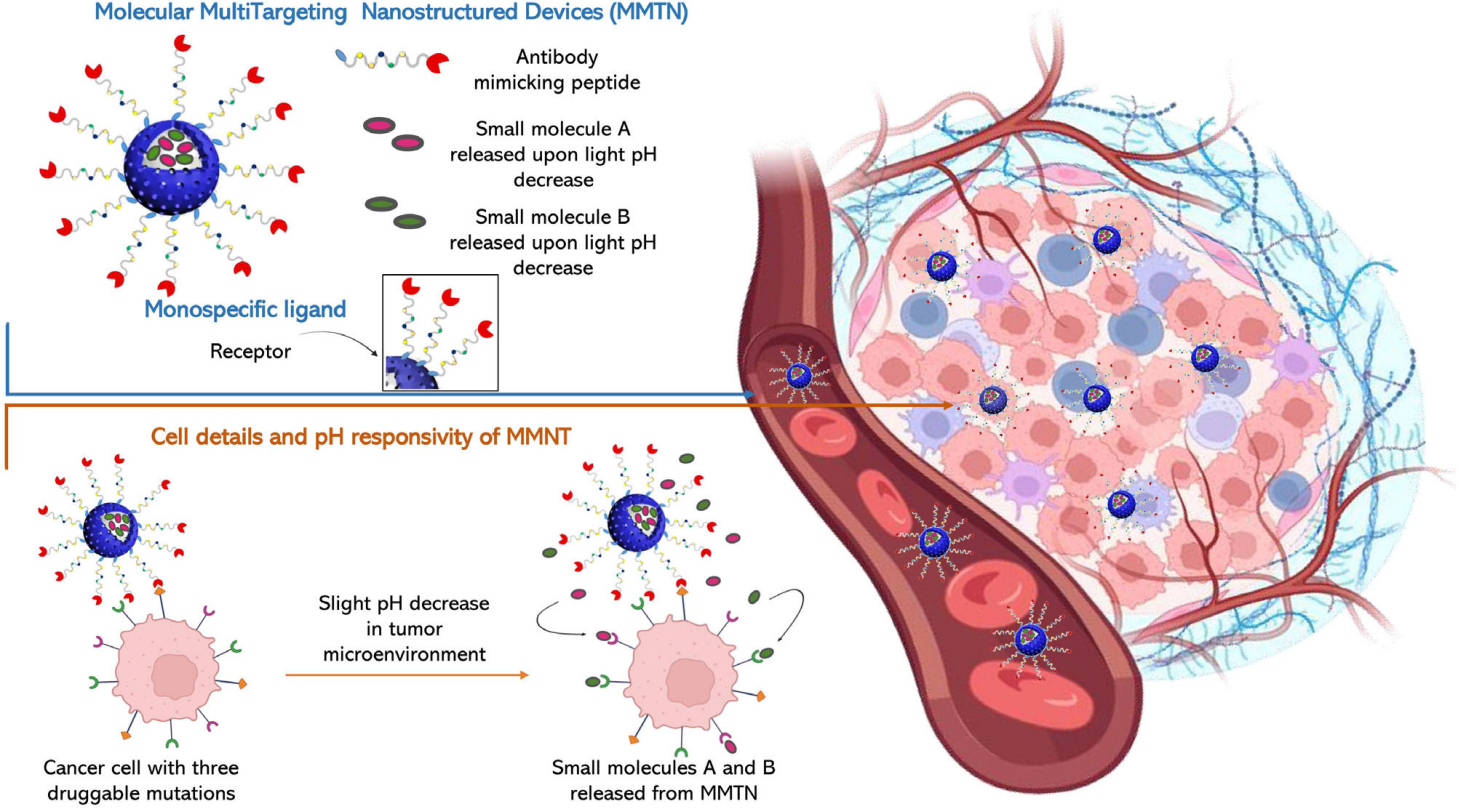
Graphical representation of the imaginary molecular multi‐targeting nanodevice (MMTN) as a possible future scenario in the field of molecular targeted therapy (MTT). For the sake of clarity, the mutual size ratio between the MMTN nanodevice and the interacting cancer cells, both in the *tumor* microenvironment (TME) and in the blood, is intentionally altered in favor of the particle that result to be magnified. For the real proportions see Figure 3.

In **Scheme** **1**, we depict a potential molecular multi‐targeting nanodevice (MMTN) that can be developed using established nanotechnological approaches. This, in our perspective, represents a plausible future scenario within the field of MTT.

The main perspective elements concerning cancer therapies, presented very recently in the literature, are reported in **Table** **3**. These analyzed papers highlight the glimpsed potential developments, ranging from nanotechnology‐related issues, also implemented by artificial intelligence, to ethical, legal, and societal considerations.

**Table 3:** Main perspectives elements in cancer therapy from the literature.

References	Possible glimpsed scenario [s]		Needs	Challenging conditions		Reported advantages
[118]	Improved nanomedicine translation and application, including exploration of alternative administration routes and delivery of biologics and non‐standard drugs.		Patient stratification for Cancer Drug development. Develop criteria for clinical trial participation. Cooperation among academics, clinicians, pharmaceutical companies, and regulatory authorities to expand nanomedicine use. Strategic planning for nanomedicine clinical trials.	Limited translation and exploitation of nanomedicine.		Enhanced Anticancer Immunity: Nan medicines can empower anticancer immunity by regulating the behavior of immune cells, making immunotherapy more effective. Multiple drug administrations. Delivery of non‐standard drugs
	Investments in modular (pro)drug and design and nanocarriers for precise and efficient drug delivery.		
	Enhance overall benefits through optimized combination therapies. Integration of nanomedicine with standard chemotherapy, co‐encapsulating multiple drugs in one formulation to enhance efficacy and responsiveness to external stimuli.		
[119]	Combining nanotechnology with cancer immunotherapy to create “nano‐immunotherapeutics” provides unique opportunities for both fields		Defining the specific medical questions in immunotherapy is crucial. For clinical translation, uniqueness and necessity outweigh abundance. Developing evaluation criteria and methods for nanotechnologies in cancer immunotherapy is key to maximizing clinical success.	Low translational rate of nanomedicine.		Immunotherapy in cancer treatment offers the potential for long term tumor inhibition or even cure, as it triggers a systemic response, and can stimulate a lasting immune memory. Protein, gene therapies and cell therapies are becoming prevalent. Integrating nanotechnology with these emerging biotechnologies maximizes their potential
[120]	Artificial Intelligence (AI) and other computational models will play an important role in the development, design, and implementation of nanotechnologies.		Diagnostic nanomaterials are used to assemble a patient‐specific profile. Integrate AI approaches to develop pattern analysis and classification algorithms for improved diagnostic and therapeutic accuracy.	Intratumor and interpatient heterogeneities pose significant challenges to the rational design of diagnostic and therapeutic platforms, as well as the analysis of their outcomes.		Improved treatment outcome. Nanomedicine design benefits from the application of AI, by optimizing material properties according to predicted interactions with the target drug, biological fluids, immune system, vasculature and cell membranes, ultimately impacting therapeutic efficacy.
	Developing a treatment protocol that combines therapies targeting multiple pathways in each patient can help overcome drug resistance and improve therapeutic efficacy.		
[121]	Translational Precision Medicine comes with a paradigm shift from a one‐size‐fits‐all to a biomarker‐guided patient‐centric medicine promoted by digital technologies and artificial intelligence (AI).		Incorporating forward and reverse translation, categorizing diseases into multi‐omics‐defined endotypes, and integrating AI‐driven R&D concepts are essential for advancing medical research. Implementing digital biomarkers and companion diagnostics is critical for improving patient care. Novel patient‐centric approaches are needed to actively engage patients in research and clinical trials. Cloud‐based data systems and platforms for interactions are required to facilitate collaboration between regulatory agencies, industry, and academia, promoting the transition to data‐driven medicine.	Ensuring feasibility of clinical trials, particularly those involving multiple center and tissue‐derived omics. Restricting the number of well‐selected clinical sites, enforcing strict standard operating procedures (SOPs), implementing cross‐site controls, and utilizing qualified analytical core facilities, are essential for robust data generation. Well‐curated biobanks are pivotal to link multi‐omics data to disease characteristics and clinical trial outcomes.		
[122]	Precision oncology will refine treatment strategies, improving outcomes and quality of life for many patients.		Refine patient selection for precision oncology. Improve the creation of decision support teams. Increase patient awareness.	–		Precision oncology medicine not only extends life but also enhances its quality compared to chemotherapy.
[123]	Nanotechnology can greatly enhance conventional cancer treatments, immune therapies, diagnostics, radiation therapies, and imaging.		Considering production costs, scalability, safety, and complexity of nanoformulations compared to potential benefits. Evaluating costs that increase with complexity of design, materials, manufacturing criteria and testing parameters.	Barriers to clinical translation include challenges related to scalability, uniformity, and regulatory guidelines.		Cost‐benefit analysis should include the long‐term quality of life impact of expensive nanomedicine solutions beyond clinical trials.
	The rapid progress of nanotechnology across different fields shows great potential for enhancing patient outcomes and quality of life.		
	Machine learning can optimize nanomedicine by analyzing patient tumor profiles and drug responses to nanoparticles.		
[124]	Nanotechnology and nanoparticles have potential to improve the efficacy of precision medicine in oncology, immunotherapy, and genome engineering.		More stratified trials are necessary. Limiting the number of patients that are eligibleto receive a medication.	Limited efficacy in broad populations, due to the vast heterogeneity of biological barriers, potentially obscuring successful treatment in smaller subgroups. High development cost for advanced NP designs, reducing the potential market size for each NP‐ based therapeutic.		Enhancing precision medicine in oncology. NP platforms effective in specific patient populations can be applied to deliver various therapeutics, including precision‐based and generic drugs. Developing one highly effective NP platform for a stratified group could lead to multiple successful clinical applications, potentially offering higher therapeutic efficacy compared to NPs developed for broad populations.
	Advanced nanoparticle design increases development costs and the financial risks linked with potential failures in clinical translation.		
	Intelligent nanoparticle design improves precision medicine by leveraging insights like patient stratification and genetic profiling for optimal selection, aiming for the ideal nanoparticle‐based solution.		
[125]	The era of personalized and precision medicine, along with its enabling technologies, offers unprecedented abilities to detect and monitor disorders.		Efficiently integrating biomarker development and potentially redesigning drug trial methodologies are key considerations. AI‐based approaches in drug development offer promising solutions to meet these challenges.	After technology validation, risks and challenges arise in healthcare economics, ethics, and data privacy.		Enhanced treatment outcomes, fewer complications, and lowered healthcare costs are key improvements.
	These technologies accurately diagnose diseases, search vast databases for potential therapies, identify biomarkers reflecting disease states, monitor disease progression through wearable technology, and optimize drug selection and doses dynamically using AI. Engineering precision medicine utilizes population‐wide data to tailor treatments to individuals.		
[126]	Directing researchers to customize nano−bio interfaces to enhance in vivo performance and facilitate clinical translation of nanomaterials/nanomedicines.		Modifying the interaction between nanomaterials and biological environments through surface and structural engineering. This includes superficial modifications through physico‐chemical methods, adjusting inherent architecture, or conducting structural engineering.	The complexity and specificity of the nano‐bio interface at the nanometer scale pose challenges.		Promote and improve clinical translation for most nanomaterials/nanomedicines.
[127]	Enhance the clinical translation efficiency in nanomedicine and personalized medicine. Personalized medicine utilizes nanomedicine to enhance binding, bioavailability, and compatibility, ensuring optimal therapeutic efficacy through controlled drug release tailored to specific targets, patients, and timing.		Improve toxicological investigations for improving the chances of success for nanomedicine. Deepen molecular and genetic disease understanding to tailor medication, reducing failure risks and enhancing patient‐specific treatment comprehension. Patient stratification.	Key challenges in nanomedicine: safety, cost, and scalability. Precision medicine, faces challenges related to ethical, social, and legal issues.		–
[128]		Development and implementation of the personalized medicine at European level concerning healthcare infrastructures and formal frameworks.	Enhance interdisciplinary and cross‐border collaboration, involving stakeholders across healthcare sectors. Progress is crucial in areas such as data protection, clinical trial strategies, health economics, and regulatory frameworks, including AI and genomic databases. Achieving innovation requires partnerships between public, private, academic, and industry sectors engaging researchers, clinicians, patients, policymakers, and society at large. Consensus among all these players can create a financially sustainable ecosystem and ethico‐legal framework supporting key developments in molecular diagnostics, data sharing, genetic counseling, and novel trial designs, among them, hybrid ones involving concomitant RVE generation.		Limited access to advanced biomarker testing and the high cost and complexity of the overall process.	The ultimate goal of precision medicine is to enhance patient outcomes, including survival rates, disease remission, and quality of life. Its cost‐effectiveness is measured by weighing the benefits against implementation costs, assessed through clinical trials, real‐world evidence, and patient‐reported outcomes.

The proposed MMTN can be developed using sustainable procedures, that we have already experimented, for the most part respecting the principles of green chemistry. Mainly water or other not extremely toxic and easily‐recoverable solvents such as ethanol or cyclohexane are employed. The synthesis of the starting architecture is carried out at room temperature without mineralizing agents and the employed surfactant is removed by water extractions for potential re‐use. Scaling up the synthesis to produce larger quantities of MSN involved the use of a 25‐fold larger reactor under the same operative conditions. The synthesis procedures, also in these conditions have exhibited their high reproducibility producing materials with identical properties. Modification reactions also occur at room temperature, typically in ethanol. Additionally, mesoporous silica can alternatively be synthesized^[^ ^109^ ^]^ and modified with stimuli‐responsive functionalities^[^ ^110^ ^]^ under MW irradiation in solvent‐free conditions. The surface characteristics of nanomaterials play a crucial role in their interactions with biological entities. Advancing knowledge and integrating nanomaterial synthesis with safety assessments will validate safer‐by‐design strategies.^[^ ^111^ ^]^

## 7 Perspectives

In this opinion article we run, not comprehensively, through the history of the target therapy for cancer. We have tried to present the different generations of therapies in a way approachable for non‐specialist readers.

Furthermore, our approach fosters broader engagement from experts across different fields, encouraging the formulation of constructive, multidisciplinary solutions that harness the added value of diverse expertise.

Navigating through the article, the increasing complexity of the analyzed needs allowing advancement in therapeutic outputs therapies is explored, reflecting the progression across different eras. Sustainable solutions nowadays and in perspective should satisfy these needs remaining able to work as a drug in the organism.

This is the era of targeted molecular therapies but single‐targeting drugs have already shown very deep limits. Multi‐targeting approaches, on the other side, have, until now, requested solutions to the chemical biology field but we think that important evolutions in this field are not possible due to the limits of multifunctional molecules to behave as a drug in the body. Apart for the potentialities of nanoimmunology^[^ ^112^ ^]^ for cancer and their combined solutions with targeted molecular therapy that fall outside the ambit of this article, we glimpse, believe, imagine that materials engineering could overcome the limits that currently complicate the development of multitargeting solutions. Our imaginary nanodevice (MMTN, Scheme 1) is the result of this vision.

## Conflict of Interest

The authors L.P., A.L., and C.M. are co‐founders of NanoSiliCal Devices, a company incorporated in Italy involved inthe development of nanotechnology‐assisted solutions for cancer therapies. The have also the role of CEO and consultants respectively in the company.
